# Goji Berries as a Potential Natural Antioxidant Medicine: An Insight into Their Molecular Mechanisms of Action

**DOI:** 10.1155/2019/2437397

**Published:** 2019-01-09

**Authors:** Zheng Feei Ma, Hongxia Zhang, Sue Siang Teh, Chee Woon Wang, Yutong Zhang, Frank Hayford, Liuyi Wang, Tong Ma, Zihan Dong, Yan Zhang, Yifan Zhu

**Affiliations:** ^1^Department of Health and Environmental Sciences, Xi'an Jiaotong-Liverpool University, Suzhou 215123, China; ^2^School of Medical Sciences, Universiti Sains Malaysia, Kota Bharu, 15200 Kelantan, Malaysia; ^3^Department of Food Science, University of Otago, Dunedin 9054, New Zealand; ^4^Department of Food Science, Faculty of Applied Sciences, Tunku Abdul Rahman University College, Kuala Lumpur 53300, Malaysia; ^5^Department of Biochemistry, Faculty of Medicine, MAHSA University, Bandar Saujana Putra, Jenjarom, 42610 Selangor, Malaysia; ^6^Jinzhou Medical University, Jinzhou 121000, China; ^7^Department of Nutrition and Dietetics, School of Biomedical and Allied Health Sciences, College of Health Sciences, University of Ghana, P. O. Box KB143, Korle-Bu, Accra, Ghana; ^8^Department of Integrative Medicine and Neurobiology, School of Basic Medical Sciences, Shanghai Medical College, Institutes of Integrative Medicine of Fudan University, Shanghai 200032, China

## Abstract

Goji berries (Lycium fruits) are usually found in Asia, particularly in northwest regions of China. Traditionally, dried goji berries are cooked before they are consumed. They are commonly used in Chinese soups and as herbal tea. Moreover, goji berries are used for the production of tincture, wine, and juice. Goji berries are high antioxidant potential fruits which alleviate oxidative stress to confer many health protective benefits such as preventing free radicals from damaging DNA, lipids, and proteins. Therefore, the aim of the review was to focus on the bioactive compounds and pharmacological properties of goji berries including their molecular mechanisms of action. The health benefits of goji berries include enhancing hemopoiesis, antiradiation, antiaging, anticancer, improvement of immunity, and antioxidation. There is a better protection through synergistic and additive effects in fruits and herbal products from a complex mixture of phytochemicals when compared to one single phytochemical.

## 1. Introduction

Goji berries (Lycium fruits) are obtained from two closely related plants, *Lycium chinense* and *Lycium barbarum*. They are usually found in Asia, particularly in northwest regions of China. *Lycium* belongs to the Solanaceae family that yields numerous foods, including some fruits that are yellow to red, ranging from potatoes and tomatoes to eggplants. Both of these *Lycium* species are generally marketed as goji berry as well as wolfberry. It is a 1-2 cm long berry, bright orange–red ellipsoid colour with a sweet and tangy flavor [1]. After harvesting in late summer–early autumn, it is sun-dried as a dried fruit.

Traditionally, dried goji berries are cooked before they are consumed. They are commonly used in Chinese soups and as herbal tea. Moreover, goji berries are used for the production of tincture, wine, and juice [2]. Many pharmacological functions related to the eyes, kidney, and liver particularly have been promoted by the consumption of goji berry in populations [3]. Goji berries are often incorporated into herb formulas. The dose of goji berries is in the range of 6-18 g. However, if goji berries are used as a single herb remedy, this dose may be insufficient. This is because the other herbs in the specific formulation may contain same components as goji berries such as polysaccharides and carotenoids. Goji berries could be used as a major component in a formulation or as a single herd. One of the recommended therapies in the treatment of atrophic gastritis is to consume twice daily with 10 g Lycium fruits each time. Besides that, 15 g of goji berries per day is considered beneficial to supply adequate zeaxanthin which is estimated at 3 mg/day as a dietary supplement for eye health [4]. A 20 g Lycium fruit in a simple tea is able to improve decreased visual perception [5]. Hence, the dosage range of goji berry alters to 15-30 grams (2- to 5-fold increases) when it is the main herd apart from that in the complex formula where the dosage range is around 6-18 g [4].

Goji berries are gradually being regarded as a functional food in many Asian countries as well as throughout Europe [3]. They also have been marketed as a health food in the western countries [6]. Goji berries recently gained a growing popularity as a “superfruit” in North America and European countries because of their potential health-promoting properties. For example, goji berries have been used to increase longevity and for the benefits to liver, kidney, and vision since ancient times [2]. Due to the rich medical properties and chemical composition, goji berry has been consumed as an important food of a health-promoting diet for hundreds of years.

## 2. Bioactive Compounds of Goji Berries

There are many bioactive compounds distinguished by high antioxidant potential in goji berries. The nutrients in goji berries are included 46% of carbohydrate, 16% of dietary fiber, 13% of protein, and 1.5% of fat. Thus, goji berries can be an excellent source of macronutrients. Micronutrients which included minerals and vitamins can be found in goji berries as well. There are studies that reported the presence of riboflavin, thiamine, nicotinic acid, and minerals such as copper, manganese, magnesium, and selenium in goji berries [7]. The bioactive compounds responsible for health benefits have been evaluated based on the macronutrients and micronutrients of goji berries. The high biological activity components in goji berries are polysaccharides, carotenoids, and phenolics [8]. These functional components are related with the health-promoting properties of goji berries.

The most important group of compounds present in goji berries is polysaccharides. Polysaccharides comprise 5–8% of dried fruits, and they are found in the water-soluble form of highly branched *L. barbarum* polysaccharides [1]. These six kinds of monosaccharides (i.e., arabinose, galactose, glucose, rhamnose, mannose, xylose, and galacturonic acid) are found in goji berries [5].

A group of carotenoids are the colour components of Lycium fruit. Carotenoids are the second highly significant group of biologically active compounds with health benefit properties present in goji berry. The total carotenoid content of different goji berries ranged from 0.03 to 0.5% of dried fruits. Being responsible for the characteristic bright and vivid orange to red colouration, the lipid soluble carotenoids occur at extremely high levels in goji berries [2]. One of the most common carotenoids found in goji berries is zeaxanthin in the form of dipalmitin zeaxanthin. In ripening goji berries, the content of zeaxanthin can reach around 77.5% of total carotenoids. Zeaxanthin palmitate (phasalien) contains 31–56% of the total carotenoids. As for now, the best natural source of dipalmitin zeaxanthin is goji berries. The fractions of beta-carotene (35.9 *μ*g/g), cryptoxanthin, and neoxanthin (72.1 *μ*g/g) are also detected in goji berry extracts [8].

Phenolic acids and flavonoids are examples of the phenolic compounds found in goji berries. Some phenolic compounds in goji berries are caffeic acid (3.73 *μ*g/g), caffeoylquinic acid (0.34 *μ*g/g), chlorogenic acid (12.4 *μ*g/g), p-coumaric acid (6.06 *μ*g/g), quercetin-diglucoside (66.0 *μ*g/g), kaempferol-3-*O*-rutinoside (11.3 *μ*g/g), and rutin (42.0 *μ*g/g) [8]. These phenolic compounds have a very high antioxidant capacity [8].  Table 1 summarises some chemical compounds found in goji berries [9].

## 3. Pharmacological Properties of Goji Berries

Goji berries have become popular over the years due to its public acceptance as a “superfood” with highly advantageous antioxidant and nutritive properties. A superfood is a “nutrient-rich” food considered to be especially beneficial for health or well-being. The carotenoid content of goji berries had been drawn a lot of attention due to its beneficial effects including antioxidant property on vision, retinopathy, and macular degeneration.

In very recent years, interest of consumers about the health benefits of different berry-type fruits, their resultant juices, and their capsules has quickly increased [10]. Berry fruits are rich in antioxidant phytochemicals [11, 12], and these antioxidants are capable of performing a number of functions. The present interest about the properties of several kinds of berries is also shown by the numerous scientific articles published in journals in the last few years. The biennial International Berry Health Benefits Symposium started in 2005 and in their latest research also focused on berry consumption in relation to human health as a key component of their symposium [13, 14]. The most extensively consumed berry-type products are derived from goji (*Lycium barbarum*), chia (*Salvia hispanica*), açaí (*Euterpe oleracea* Martius), jujuba (*Ziziphus jujuba*), pomegranate (*Punica granatum*), and mangosteen (*Garcinia mangostana*) [10]. The dietary intake of berry fruits has been shown to have a positive impact on human health, performance, and disease [15–24]. All these fruits support the immune system and are rich in nutrients. Overall, they have a significant concentration of phytosterols, monounsaturated fats, antioxidants, essential amino acids, trace minerals, dietary fiber, and fat- and water-soluble vitamins [25].

Goji berry polysaccharides, for instance, are a well-known traditional Chinese medicine and tonic food for many years. In connection with it health benefits, *Lycium barbarum* polysaccharides (LBPs) are one of the most valuable functional components [5, 26]. In recent years, *L. barbarum* is being used not only in China but also worldwide as a health dietary supplement in several forms including juice and tea [27]. Consuming products made from *L. barbarum* might help to decrease blood lipid concentration, promote fertility, and improve immunity [17, 19, 27–29].

### 3.1. Vision-Protective Effect

The mixture of highly branched polysaccharides and proteoglycans in LBPs has been reported to exert ocular neuroprotective effects [30, 31]. Goji berries, which contain a specific profile of carotenoid species [2, 32], have high carotenoid metabolites, with zeaxanthin making up almost 60% of the total carotenoids in the fruit [33]. Carotenoids are main natural pigments accountable for the yellow, red, and orange colours of many types of fruits and vegetables [34]. They also have many biological actions including the pro-vitamin A's antioxidant activity.

LBPs are the active components which may improve visual function. Chu et al. [28] reported an animal study investigating the effects of LBPs (1 mg/kg) on localised changes of rats' retinal function in a partial optic nerve transection (PONT) model. The multifocal electroretinograms (mfERG) were obtained from Sprague-Dawley rats. One week later, a substantial decrease of major positive component (P1) and photopic negative response (PhNR) amplitudes of mfERG were detected in all retinal regions. Feeding with LBPs prior to PONT preserved the functions of retina. All mfERG responses were reported to be within the normal range in the superior retina, and most of the inferior retinal responses were considerably increased at week 4. The retina ventral part had secondary degeneration which affected the ganglion cell layer and outer retina. LBPs caused alterations to the functional reduction caused by PONT by regulating the signal from the outer retina. Zhu et al. reported that LBPs inhibited the N-methyl-N-nitrosourea-induced rat photoreceptor cell apoptosis [35]. In addition, LBPs also protected the retinal structure by regulating the expressions of caspase and PARP [35].

The protective characteristics of goji berry extracts on retina cells have been shown in the early stage of the retina degeneration in both human and animal studies [1]. Consumption of dietary *L. barbarum* has been shown to be retinoprotective. A study by Yu et al. in 2013 showed that 1% (kcal) wolfberry upregulated carotenoid metabolic genes of zeaxanthin and luteolin and also improved the biogenesis of mitochondria in the retina of db/db diabetic mice [36]. It has been suggested that inhibited expression of these zeaxanthin and luteolin-metabolizing genes can cause hyperglycaemia, which increases the risk of retinopathy [1].

Using Royal College of Surgeons (RCS) rats as a hereditary retinal dystrophy model, Ni et al. examined the potential neuroprotective effects of aqueous extract of dried *L. barbarum* [37]. The results indicated that the aqueous extract of dried *L. barbarum* might possess a neuroprotective activity on the retinal tissue of RCS rats at the initial stage by hindering apoptosis involving caspase-2 protein and protecting photoreceptors [37]. Prior studies had established that apoptosis is the dominant mechanism of photoreceptor degeneration in RCS rats [38, 39]. The contribution of polysaccharide fractions of *L. barbarum* to the prevention of glaucoma was further demonstrated on the retinal ganglion cells (RGC) in rats with high intraocular pressure (IOP), indicating the neuroprotective effect of *L. barbarum* [40]. Further research work by Tang et al. [41] and Hu et al. [42] also established the protective effect of *L. barbarum* on diabetic retinal injury.

Goji berries have also been shown to exhibit macular benefits in a randomized controlled study of healthy elderly participants [43]. It was observed that after 90 days of daily dietary supplementation with 13.7 g lacto-wolfberry (LWB) (a proprietary milk-based formulation of goji berry) elevated plasma antioxidant and zeaxanthin levels group, by 26% and 57%, respectively, in supplemented subjects [43]. It is also suggested that taurine, a nonessential free amino acid in goji extracts, may hinder the diabetic retinopathy progress through elevated cAMP levels and enhanced PPAR-*γ* activity in retinal cells [44]. Taurine is found abundantly in goji. Goji powder extracted with methanol contains 10.7 ± 0.1% taurine (*w*/*w*). Elevated cAMP levels have been known as protective against the dysfunction of the endothelial barrier [45, 46]. Results from Pavan et al. [47] strongly suggested that in high glucose-treated cells, elevated cAMP concentrations mediate the impairment of the epithelial barrier and goji berries could be used to achieve their reversal. The protective property of *L. barbarum* extract was also confirmed by Shen et al. using human retina neuron cells [48].

### 3.2. Lipid-Lowering Effect

The lipid-lowering health benefit of LBP and its purified constituents have been demonstrated in animals with limited clinical studies in humans. Besides having antioxidant activity in vitro [8, 49, 50] and in vivo [49, 51], they have also shown to have the ability to lower the blood lipid concentrations of alloxan-induced diabetic rabbits [7] and mice fed by high-fat diet (HFD) [52]. Ming et al.'s research showed that abnormal lipid peroxidation parameters were returned to near normal level and lipid peroxidation accumulation was inhibited after administrating LPS to mice fed on HFD. This suggests that LBP seems to play an imperative role in lipid metabolism [52]. The results were consistent with previous findings, where mice and rats fed with polysaccharide fractions supplemented with HFD were characterized by lowered concentration of total cholesterol, LDL-cholesterol, and triglycerides and increased concentration of HDL-cholesterol compared to mice and rats on high-fat diets without polysaccharide fractions [53–55]. The evaluation of the lipid profile of diabetic mice and rats fed on goji extract also showed the same results compared with the diabetic controls [1, 7]. However, clinical studies on the lipid-lowering properties of goji berries were limited and almost exclusively performed in China. More so, original data are hardly accessible, and studies were mostly small-sized and may not have been adequately controlled. A study of 25 Chinese subjects aged 64-80 years had their blood lipid peroxides significantly decreased by 65% after 10 days of ingestion of 50 g/d dry goji berries [56, 57]. However, the small size of the study (*N* = 25) and the subjectivity of most parameters must be critically pointed out. An in vivo investigation of the effects of serum LBP-standardized *L. barbarum preparation* (GoChi) in a randomized, double-blind, placebo-controlled clinical study involving 50 Chinese healthy adults aged 55-72 years showed a significant decrease in lipid peroxidation (shown by lower concentrations of malondialdehyde (MDA)) by 8.7% and 6.0% pre-intervention and post-intervention in the GoChi group compared with the placebo group, respectively. This was after they were given GoChi or placebo (120 mL/d) for 30 days [26].

### 3.3. Hypoglycaemic Effect

Diabetes mellitus is characterized by abnormally high levels of blood glucose, and it is also known as hyperglycaemia [58]. Due to the high cost and adverse side effects of many oral hypoglycaemic agents, the exploration and discovery of safer and more effective substitutes have become very important and significant. This has led to the investigation for hypoglycaemic activity in other more traditionally edible food sources such as goji berries which have been shown to have a hypoglycaemic effect in cell and animal studies [1]. A cell experiment on hypoglycaemic effects for instance proved that LBP3b (an extraction from *L. barbarum* fruit) showed a concentration-dependent effect on glucose uptake [59]. Male Wistar HFD-STZ-induced diabetic rats administered with immunoglobulin (Ig) LBP and LBP-IV once daily for 4 continuous weeks and treated with LBP (100 mg/kg) and LBP-IV (200, 100, and 50 mg/kg) after showed significantly decreased concentrations of HbA1 and blood glucose of diabetic rats compared to the diabetic control group [60]. Alloxan-induced diabetic rabbits fed with crude LBP and purified polysaccharide fraction (LBP-X) from *L. barbarum* for 10 days also showed a significant reduction in blood glucose level [7, 61]. Similar results were observed after a 28-day treatment in alloxan-induced diabetic mice with LBP [62–64]. This was consistent with Zou et al.'s [65] findings where the rat insulinoma cell line was used. Very limited or no clinical human studies exist, however.

### 3.4. Allergic and Anaphylactic Reactions

Monzon-Ballarin et al. [66] described two clinical cases who reported allergic symptoms after goji berry ingestion. The patients had a positive skin prick test and a detection of specific immunoglobulin (Ig) E to goji berry. An analysis of the allergenic profile of the two patients showed a 9 kDa band, suggesting that the corresponding protein might be related to lipid transfer proteins (LTPs). Larramendi et al. [67] further reported a study involving 31 subjects in Spain. The subjects included five patients reporting allergenic symptoms on intake of goji berries, six tolerating the berries, and 20 never having eaten goji berries. All subjects underwent skin prick tests with goji berries, as well as with peach peel and plant food panallergens as biomarkers of cross-reactivity between unrelated foods. They reported that the skin tests to goji berries were positive in 24 subjects (77%). Positivity to goji berries was related with positivity to peach peel and to the panallergen-nonspecific LTPs.

### 3.5. Anticancer, Antitumour, Immunostimulatory, and Modulatory Effects

Goji berries have been utilised in traditional Chinese medicine to prevent the onset and progression of cancer for so many years, due to its rich phytochemical and antioxidant composition [1]. Some of its ingredients might have a better therapeutic effect on cancer than other foods. Hsu et al. [68] have reported that the *L. barbarum* carotenoid nanoemulsion was more effective in inhibiting HT-29 cancer cells as compared to that of the carotenoid extract. Furthermore, both nanoemulsion and extract could upregulate p53 and p21 expression and downregulate CDK1, CDK2, cyclin A, and cyclin B expression and arrest the cell cycle at G2/M. Moreover, attributing to most of the biological effects of the fruits including anticancer, antitumour, and immunomodulatory and properties, goji berries are unusually rich in water-soluble peptide-conjugated polysaccharides (i.e., LBPs) [69–71]. They have the ability to enhance or potentiate the host defence mechanisms in a way to inhibit tumour growth without harming the host. Research work conducted by Tang et al. [41] and Gan et al. [69] established that compounds in goji berries have proapoptotic and antiproliferative activity against cancer cells.

### 3.6. Neurological Protective Effect

The neurological protective effect of goji berries has been demonstrated in an experimental study including human clinical trial. Glutamate has been shown to be excitotoxic and is being implicated in many neurodegenerative diseases including Parkinson's disease and Alzheimer's disease [61, 72]. Thus, reduction of glutamate toxicity is considered a therapeutic strategy for those neurodegenerative diseases.

A study by Yang et al. [73] showed that LBP pretreatment significantly improved neurological deficits by decreasing the infarct size, hemispheric swelling, and water content in an experimental stroke model C57BL/6N male mice fed with either vehicle (PBS) or LBP (1 or 10 mg/kg) daily for 7 days, indicating the neuroprotective effect of LBP. LBP again improved the survival rate and promoted the growth of mixed cultured retinal ganglion cells, from neonatal Sprague-Dawley rats [74]. The first double-blind randomized control study performed outside China to assess the general effects of goji juice (GoChi™) in young healthy adults concluded that consumption of GoChi™ for 14 days improved neurological performance generally [75]. It should be noted however that assessment of most parameters was subjective and the sample size was small in this study (*N* = 34).

### 3.7. Cardiovascular Protective Effect

In an experiment to investigate the role of LBP in the reduction of myocardial injury in ischemia/reperfusion among rats, the rat heart LBP significantly reduced the myocardium Bax-positive rate; also, through dose-dependent methods, the apoptosis of myocardial cell and increase in Bcl-2 positive rate suggest that LBP can prevent further development and deterioration of CVD [76]. Regarding the effects on renal vascular tension of LBP, Jia et al. [77] tested the one-clip hypertension model among rats with hypertension. It was observed that compared to rats not treated for hypertension, in isolated aortic rings of LBP-treated rats, the reduced phenylephrine contraction was observed, causing that LBP-treated rats were significantly prevented from elevated blood pressure. Another experiment was that rats with hyperlipidemia were administered to take different concentrations (1 g/kg to 4 g/kg) of *L. barbarum* decoction for 10 consecutive days by gastric perfusion. The authors reported that in the serum and liver of rats, total cholesterol and triglyceride levels were reduced; also, the level of serum low-density lipoprotein- (LDL-) C was decreased [5, 78]. A similar result was observed in a study by Luo et al. [7]. LBPs lowered serum total cholesterol and triglyceride levels; meanwhile, the high-density lipoprotein (HDL) cholesterol level was increased after a 10-day treatment among rabbits.

### 3.8. Antiaging Effects

In a recent review, Gao et al. [79] have discussed the various components contributing to the antiaging properties of *L. barbarum*. These notable components are LBPs, betaine, *β*-carotene, zeaxanthin, 2-O-*β*-D-glucopyranosyl-L-ascorbic acid (AA-2*β*G), and flavanoids [79]. *L. barbarum* contains betaine (a natural amino-acid). The Lycium Chinese Miller fruit extract containing betaine has been shown to mitigate carbon tetrachloride- (CCl4-) induced hepatic injury by increasing antioxidative activity and lowering inflammatory mediators such as COX-1/COX-2 and iNOS. Histopathological examination was employed to confirm the ameliorative effects of the extract and betaine [80].

Betaine has been shown to be an anti-inflammation agent associated with colon carcinogenesis. It also has been shown to possess a tumour-preventing effect on colitis-associated cancer in mice induced by azoxymethane. Administration with betaine significantly lowered the incidence of tumour formation with downregulation of inflammation. Treatment with betaine also inhibited the production of the ROS and GSSG level in colonic mucosa and inhibited inflammatory cytokines including IL-6, iNOS, TNF-*α*, and COX-2 [81]. Betaine has been shown to have preventive effects on ultraviolet B (UVB) irradiation-induced skin damage in mice. UVB is a common kind of free radical that can cause extrinsic aging, such as skin aging. Betaine has been proved to reduce photodamage caused by UVB irradiation. Betaine can be used to suppress the formation of UVB-induced wrinkle and collagen damage by inhibiting the extracellular signal-regulated kinase (ERK), protein kinase (MEK), and matrix metalloproteinase 9 (MMP-9) [82].

### 3.9. Adverse Effects of Goji Berries

Apart from the allergic and anaphylactic reactions, other side effects that consumers should be aware of are to be mentioned. These include the presence of organic toxic substances and risk of interactions with other prescriptions besides allergy. Atropine, a toxic alkaloid, is naturally present in goji berry. The content was reported to be at toxic level. In a further work by Adam and co-workers, the atropine concentration in eight samples of goji berries using HPLC-MS was found to be maximally 19 ppb (*w*/*w*). Therefore, its content is far below toxic levels (Adam et al., 2006).

Patients who experienced interactions between goji berries and warfarin have been described in three published case reports. Warfarin is prescribed as a common anticoagulation therapy. The international normalized ratio (INR) was observed to elevate in patients after drinking goji tea [83]. Increased bleeding from the rectum and nose was observed in another patient who drank goji berry juice [84]. Most recently, a study by Zhang et al. reported that an elderly man taking a prolonged maintenance dose of warfarin after drinking goji berry wine experienced an increased international normalized ratio (INR) with associated bleeding [85]. Other possible interactions between goji berries and prescription medications are still unknown. It is important to take into consideration the possible risks of taking goji berries in individuals taking medications with a narrow therapeutic index.

Arroyo-Martinez et al. described a case report of toxic hepatitis related to the use of goji. The symptoms reported included nonbloody diarrhea, asthenia, and colic abdominal pain. The patient had a mild mucocutaneous jaundice and a generalized erythematous and pruriginous maculopapular rash. The patient consumed goji berry tea 3 times a day [86]. The liver function tests were elevated. Goji berries have been shown to modulate the expression of CYP2C9 and CYP2E1 and have an immunomodulatory property [2]. However, another possible change in goji composition is contamination, during its production and post-marketing. Thus, the toxic side effects of post-marketing surveillance are another area of concern.

## 4. Conclusion

Similar to other plants [87–91], goji berries are a high antioxidant potential fruits which alleviate oxidative stress to confer many health protective benefits such as preventing free radicals from damaging DNA, lipids, and proteins. There is a better protection through synergistic and additive effects in fruits and herbal products from a complex mixture of phytochemicals than from a single phytochemical. The health benefits of goji berries include enhancing hemopoiesis, antiradiation, antiaging, anticancer, improvement of immunity, and antioxidation.

## Figures and Tables

**Table 1:** Some chemical compounds of goji berries.

Composition	
Moisture (%)	10.3
Crude protein (%)	8.9
Crude oil (%)	4.1
Fiber (%)	7.3
Total phenol (mg GAE/100 mL)	3.4
Antioxidant activity (%)	20.8
Myristic acid (%)	0.1
Stearic acid (%)	2.9
Palmitic acid (%)	8.2
Arachidic acid (%)	1.8
Oleic acid (%)	21.7

## References

[1] Kulczyński B., Gramza-Michałowska A. (2016). Goji berry (Lycium barbarum): composition and health effects–a review. *Polish Journal of Food and Nutrition Sciences*.

[2] Potterat O. (2010). Goji (*Lycium barbarum* and *L. chinense*): phytochemistry, pharmacology and safety in the perspective of traditional uses and recent popularity. *Planta Medica*.

[3] Cheng J., Zhou Z.-W., Sheng H.-P. (2015). An evidence-based update on the pharmacological activities and possible molecular targets of *Lycium barbarum* polysaccharides. *Drug Design, Development and Therapy*.

[4] Cheng C. Y., Chung W. Y., Szeto Y. T., Benzie I. F. F. (2007). Fasting plasma zeaxanthin response to Fructus barbarum L. (wolfberry; Kei Tze) in a food-based human supplementation trial. *British Journal of Nutrition*.

[5] Amagase H., Farnsworth N. R. (2011). A review of botanical characteristics, phytochemistry, clinical relevance in efficacy and safety of Lycium barbarum fruit (goji). *Food Research International*.

[6] Xin T., Yao H., Gao H. (2013). Super food Lycium barbarum (Solanaceae) traceability via an internal transcribed spacer 2 barcode. *Food Research International*.

[7] Luo Q., Cai Y., Yan J., Sun M., Corke H. (2004). Hypoglycemic and hypolipidemic effects and antioxidant activity of fruit extracts from Lycium barbarum. *Life Sciences*.

[8] Wang C., Chang S., Inbaraj B. S., Chen B. (2010). Isolation of carotenoids, flavonoids and polysaccharides from *Lycium barbarum* L. and evaluation of antioxidant activity. *Food chemistry*.

[9] Endes Z., Uslu N., Özcan M. M., Er F. (2015). Physico-chemical properties, fatty acid composition and mineral contents of goji berry (*Lycium barbarum* L.) fruit. *Journal of Agroalimentary Processes and Technologies*.

[10] Llorent-Martínez E., Fernández-de Córdova M., Ortega-Barrales P., Ruiz-Medina A. (2013). Characterization and comparison of the chemical composition of exotic superfoods. *Microchemical Journal*.

[11] Lachman J., Orsák M., Pivec V. (2000). Antioxidant contents and composition in some vegetables and their role in human nutrition. *Zahradnictví (Horticultural Science)*.

[12] Lachman J., Orsák M., Pivec V. (2000). Antioxidant contents and composition in some fruits and their role in human nutrition. *Zahradnictví (Horticultural Science)*.

[13] Seeram N. P. (2008). Berry fruits: compositional elements, biochemical activities, and the impact of their intake on human health, performance, and disease. *Journal of Agricultural and Food Chemistry*.

[14] Seeram N. P. (2010). Recent trends and advances in berry health benefits research. *Journal of Agricultural and Food Chemistry*.

[15] Xie J. H., Liu X., Shen M. Y. (2013). Purification, physicochemical characterisation and anticancer activity of a polysaccharide from Cyclocarya paliurus leaves. *Food Chemistry*.

[16] Xie J. H., Zhang F., Wang Z. J., Shen M. Y., Nie S. P., Xie M. Y. (2015). Preparation, characterization and antioxidant activities of acetylated polysaccharides from Cyclocarya paliurus leaves. *Carbohydrate Polymers*.

[17] Huang D.-F., Tang Y.-F., Nie S.-P., Wan Y., Xie M.-Y., Xie X.-M. (2009). Effect of phenylethanoid glycosides and polysaccharides from the seed of *Plantago asiatica* L. on the maturation of murine bone marrow-derived dendritic cells. *European Journal of Pharmacology*.

[18] Changbo D., Zhaojun S. (2012). Supplementation of Lycium barbarum polysaccharides protection of skeletal muscle from exercise-induced oxidant stress in mice. *African Journal of Pharmacy and Pharmacology*.

[19] Xu X., Shan B., Liao C. H., Xie J. H., Wen P. W., Shi J. Y. (2015). Anti-diabetic properties of Momordica charantia L. polysaccharide in alloxan-induced diabetic mice. *International Journal of Biological Macromolecules*.

[20] Chin Y.-P., Chang S.-F., Tseng C.-C., Chen M.-C. (2014). Escherichia coli capsular polysaccharide synthesis, antibiotic susceptibility, and red blood cell agglutination. *Journal of Experimental & Clinical Medicine*.

[21] Xie J.-H., Shen M.-Y., Xie M.-Y. (2012). Ultrasonic-assisted extraction, antimicrobial and antioxidant activities of *Cyclocarya paliurus* (Batal.) Iljinskaja polysaccharides. *Carbohydrate Polymers*.

[22] Al-Reza S. M., Yoon J. I., Kim H. J., Kim J. S., Kang S. C. (2010). Anti-inflammatory activity of seed essential oil from Zizyphus jujuba. *Food and Chemical Toxicology*.

[23] Liu C. J., Lin J. Y. (2012). Anti-inflammatory and anti-apoptotic effects of strawberry and mulberry fruit polysaccharides on lipopolysaccharide-stimulated macrophages through modulating pro-/anti-inflammatory cytokines secretion and Bcl-2/Bak protein ratio. *Food and Chemical Toxicology*.

[24] Zhang H., Ma Z. F., Luo X., Li X. (2018). Effects of mulberry fruit (*Morus alba* L.) consumption on health outcomes: a mini-review. *Antioxidants*.

[25] Jeszka-Skowron M., Zgola-Grzeskowiak A., Stanisz E., Waskiewicz A. (2017). Potential health benefits and quality of dried fruits: goji fruits, cranberries and raisins. *Food Chemistry*.

[26] Amagase H., Sun B., Borek C. (2009). Lycium barbarum (goji) juice improves in vivo antioxidant biomarkers in serum of healthy adults. *Nutrition Research*.

[27] Xie J.-H., Tang W., Jin M.-L., Li J.-E., Xie M.-Y. (2016). Recent advances in bioactive polysaccharides from Lycium barbarum L., Zizyphus jujuba Mill, Plantago spp., and Morus spp.: structures and functionalities. *Food Hydrocolloids*.

[28] Chu P. H., Li H. Y., Chin M. P., So K. F., Chan H. H. (2013). Effect of lycium barbarum (wolfberry) polysaccharides on preserving retinal function after partial optic nerve transection. *PLoS One*.

[29] Liu W., Liu Y., Zhu R. (2016). Structure characterization, chemical and enzymatic degradation, and chain conformation of an acidic polysaccharide from Lycium barbarum L. *Carbohydrate Polymers*.

[30] Li S. Y., Yang D., Yeung C. M. (2011). Lycium barbarum polysaccharides reduce neuronal damage, blood-retinal barrier disruption and oxidative stress in retinal ischemia/reperfusion injury. *PLoS One*.

[31] Mi X. S., Feng Q., Lo A. C. (2012). Protection of retinal ganglion cells and retinal vasculature by Lycium barbarum polysaccharides in a mouse model of acute ocular hypertension. *PLoS One*.

[32] Bondia-Pons I., Savolainen O., Törrönen R., Martinez J. A., Poutanen K., Hanhineva K. (2014). Metabolic profiling of goji berry extracts for discrimination of geographical origin by non-targeted liquid chromatography coupled to quadrupole time-of-flight mass spectrometry. *Food Research International*.

[33] Inbaraj B. S., Lu H., Hung C. F., Wu W. B., Lin C. L., Chen B. H. (2008). Determination of carotenoids and their esters in fruits of Lycium barbarum Linnaeus by HPLC-DAD-APCI-MS. *Journal of Pharmaceutical and Biomedical Analysis*.

[34] Zhang Q., Chen W., Zhao J., Xi W. (2016). Functional constituents and antioxidant activities of eight Chinese native goji genotypes. *Food Chemistry*.

[35] Zhu Y., Zhao Q., Gao H., Peng X., Wen Y., Dai G. (2016). Lycium barbarum polysaccharides attenuates N-methy-N-nitrosourea-induced photoreceptor cell apoptosis in rats through regulation of poly (ADP-ribose) polymerase and caspase expression. *Journal of Ethnopharmacology*.

[36] Yu H., Wark L., Ji H. (2013). Dietary wolfberry upregulates carotenoid metabolic genes and enhances mitochondrial biogenesis in the retina of db/db diabetic mice. *Molecular Nutrition & Food Research*.

[37] Ni T., Wei G., Yin X., Liu X., Liu D. (2013). Neuroprotective effect of Lycium barbarum on retina of Royal College of Surgeons (RCS) rats: a preliminary study. *Folia Neuropathologica*.

[38] Travis G. H. (1998). Mechanisms of cell death in the inherited retinal degenerations. *American Journal of Human Genetics*.

[39] Tso M. O., Zhang C., Abler A. S. (1994). Apoptosis leads to photoreceptor degeneration in inherited retinal dystrophy of RCS rats. *Investigative Ophthalmology & Visual Science*.

[40] Chiu K., Zhou Y., Yeung S. C. (2010). Up-regulation of crystallins is involved in the neuroprotective effect of wolfberry on survival of retinal ganglion cells in rat ocular hypertension model. *Journal of Cellular Biochemistry*.

[41] Tang L., Zhang Y., Jiang Y. (2011). Dietary wolfberry ameliorates retinal structure abnormalities in db/db mice at the early stage of diabetes. *Experimental Biology and Medicine*.

[42] Hu C. K., Lee Y. J., Colitz C. M., Chang C. J., Lin C. T. (2012). The protective effects of Lycium barbarum and Chrysanthemum morifolum on diabetic retinopathies in rats. *Veterinary Ophthalmology*.

[43] Bucheli P., Vidal K., Shen L. (2011). Goji berry effects on macular characteristics and plasma antioxidant levels. *Optometry and Vision Science*.

[44] Song M. K., Salam N. K., Roufogalis B. D., Huang T. H. W. (2011). Lycium barbarum (goji berry) extracts and its taurine component inhibit PPAR-*γ*-dependent gene transcription in human retinal pigment epithelial cells: possible implications for diabetic retinopathy treatment. *Biochemical Pharmacology*.

[45] Sayner S. L., Alexeyev M., Dessauer C. W., Stevens T. (2006). Soluble adenylyl cyclase reveals the significance of cAMP compartmentation on pulmonary microvascular endothelial cell barrier. *Circulation Research*.

[46] Fischmeister R. (2006). Is cAMP good or bad?: depends on where it’s made. *Circulation Research*.

[47] Pavan B., Capuzzo A., Forlani G. (2014). High glucose-induced barrier impairment of human retinal pigment epithelium is ameliorated by treatment with goji berry extracts through modulation of cAMP levels. *Experimental Eye Research*.

[48] Shen Z. J., Wang J. J., Li G. L. (2012). Effect of extract of *Lycium barbarum* L. on adult human retinal nerve cells. *Zhonghua Yan Ke Za Zhi*.

[49] Liang B., Jin M., Liu H. (2011). Water-soluble polysaccharide from dried Lycium barbarum fruits: isolation, structural features and antioxidant activity. *Carbohydrate Polymers*.

[50] Ke M., Zhang X.-J., Han Z.-H. (2011). Extraction, purification of Lycium barbarum polysaccharides and bioactivity of purified fraction. *Carbohydrate Polymers*.

[51] Xiao J., Liong E. C., Ching Y. P. (2012). *Lycium barbarum* polysaccharides protect mice liver from carbon tetrachloride-induced oxidative stress and necroinflammation. *Journal of Ethnopharmacology*.

[52] Ming M., Guanhua L., Zhanhai Y., Guang C., Xuan Z. (2009). Effect of the Lycium barbarum polysaccharides administration on blood lipid metabolism and oxidative stress of mice fed high-fat diet in vivo. *Food Chemistry*.

[53] Li X. M., Ma Y. L., Liu X. J. (2007). Effect of the Lycium barbarum polysaccharides on age-related oxidative stress in aged mice. *Journal of Ethnopharmacology*.

[54] Cui B., Liu S., Lin X. (2011). Effects of Lycium barbarum aqueous and ethanol extracts on high-fat-diet induced oxidative stress in rat liver tissue. *Molecules*.

[55] Pai P. G., Habeeba P. U., Ullal S., Shoeb P. A., Pradeepti M., Ramya K. (2013). Evaluation of hypolipidemic effects of Lycium barbarum (goji berry) in a murine model. *Journal of natural remedies*.

[56] Li W., Dai S. Z., Ma W., Gao L. (1991). Effects of oral administration of wolfberry on blood superoxide dismutase (SOD), hemoglobin (Hb) and lipid peroxide (LPO) levels in old people. *Chinese Traditional and Herbal Drugs*.

[57] Burke D., Smidt C., Vuong L. (2005). Momordica cochinchinensis, Rosa roxburghii, wolfberry, and sea buckthorn-highly nutritional fruits supported by tradition and science. *Current Topics in Nutraceutical Research*.

[58] Amos A. F., McCarty D. J., Zimmet P. (1997). The rising global burden of diabetes and its complications: estimates and projections to the year 2010. *Diabetic Medicine*.

[59] Tang H.-L., Chen C., Wang S.-K., Sun G.-J. (2015). Biochemical analysis and hypoglycemic activity of a polysaccharide isolated from the fruit of Lycium barbarum L. *International Journal of Biological Macromolecules*.

[60] Zhao R., Jin R., Chen Y., Han F.-M. (2015). Hypoglycemic and hypolipidemic effects of *Lycium barbarum* polysaccharide in diabetic rats. *Chinese herbal medicines*.

[61] Jin M., Huang Q., Zhao K., Shang P. (2013). Biological activities and potential health benefit effects of polysaccharides isolated from Lycium barbarum L. *International Journal of Biological Macromolecules*.

[62] Jing L., Cui G., Feng Q., Xiao Y. (2009). Evaluation of hypoglycemic activity of the polysaccharides extracted from *Lycium Barbarum*. *African Journal of Traditional, Complementary, and Alternative Medicines*.

[63] Jing L., Yin L. (2010). Antihyperglycemic activity of polysaccharide from Lycium barbarum. *Journal of Medicinal Plants Research*.

[64] Zhou Z., Jing L., Cui G., Feng Q., Xiao Y. (2009). Effects of polysaccharide from Lycium barbarum in alloxan-induced diabetic mice. *African Journal of Biotechnology*.

[65] Zou S., Zhang X., Yao W., Niu Y., Gao X. (2010). Structure characterization and hypoglycemic activity of a polysaccharide isolated from the fruit of Lycium barbarum L. *Carbohydrate Polymers*.

[66] Monzon Ballarin S., Lopez-Matas M. A., Saenz Abad D., Perez-Cinto N., Carnes J. (2011). Anaphylaxis associated with the ingestion of goji berries (Lycium barbarum). *Journal of Investigational Allergology & Clinical Immunology*.

[67] Larramendi C. H., Garcia-Abujeta J. L., Vicario S. (2012). Goji berries (*Lycium barbarum*): risk of allergic reactions in individuals with food allergy. *Journal of Investigational Allergology & Clinical Immunology*.

[68] Hsu H. J., Huang R. F., Kao T. H., Inbaraj B. S., Chen B. H. (2017). Preparation of carotenoid extracts and nanoemulsions from *Lycium barbarum* L. and their effects on growth of HT-29 colon cancer cells. *Nanotechnology*.

[69] Gan L., Hua Zhang S., Liang Yang X., Bi Xu H. (2004). Immunomodulation and antitumor activity by a polysaccharide-protein complex from Lycium barbarum. *International Immunopharmacology*.

[70] Gan L., Zhang S. H., Liu Q., Xu H. B. (2003). A polysaccharide-protein complex from Lycium barbarum upregulates cytokine expression in human peripheral blood mononuclear cells. *European Journal of Pharmacology*.

[71] Ooi V. E., Liu F. (2000). Immunomodulation and anti-cancer activity of polysaccharide-protein complexes. *Current Medicinal Chemistry*.

[72] Ho Y.-S., Yu M.-S., Yik S.-Y., So K.-F., Yuen W.-H., Chang R. C.-C. (2009). Polysaccharides from wolfberry antagonizes glutamate excitotoxicity in rat cortical neurons. *Cellular and Molecular Neurobiology*.

[73] Yang D., Li S.-Y., Yeung C.-M. (2012). Lycium barbarum extracts protect the brain from blood-brain barrier disruption and cerebral edema in experimental stroke. *PLoS One*.

[74] Yang M., Gao N., Zhao Y., Liu L.-X., Lu X.-J. (2011). Protective effect of Lycium barbarum polysaccharide on retinal ganglion cells *in vitro*. *International Journal of Ophthalmology*.

[75] Amagase H., Nance D. M. (2008). A randomized, double-blind, placebo-controlled, clinical study of the general effects of a standardized Lycium barbarum (goji) juice, GoChi. *Journal of Alternative and Complementary Medicine*.

[76] Lu S. P., Zhao P. T. (2010). Chemical characterization of Lycium barbarum polysaccharides and their reducing myocardial injury in ischemia/reperfusion of rat heart. *International Journal of Biological Macromolecules*.

[77] Jia Y. X., Dong J. W., Wu X. X., Ma T. M., Shi A. Y. (1998). The effect of lycium barbarum polysaccharide on vascular tension in two-kidney, one clip model of hypertension. *Sheng Li Xue Bao*.

[78] Wang D., Xiao Y., Xu Z. (1998). The dose-effect relation in Gou Qi Zi’s effect of counteracting experimental hyperlipidemia and liver lipid peroxidation. *Journal of Applied Integrated Medicine*.

[79] Gao Y., Yifo W., Yuqing W., Fang G., Zhigang C. (2017). Lycium barbarum: a traditional Chinese herb and a promising anti-aging agent. *Aging and Disease*.

[80] Ahn M., Park J. S., Chae S. (2014). Hepatoprotective effects of Lycium chinense Miller fruit and its constituent betaine in CCl4-induced hepatic damage in rats. *Acta Histochemica*.

[81] Kim D. H., Sung B., Kang Y. J. (2014). Anti-inflammatory effects of betaine on AOM/DSSinduced colon tumorigenesis in ICR male mice. *International Journal of Oncology*.

[82] Im A. R., Lee H. J., Youn U. J., Hyun J. W., Chae S. (2016). Orally administered betaine reduces photodamage caused by UVB irradiation through the regulation of matrix metalloproteinase-9 activity in hairless mice. *Molecular Medicine Reports*.

[83] Leung H., Hung A., Hui A. C., Chan T. Y. (2008). Warfarin overdose due to the possible effects of *Lycium barbarum* L. *Food and Chemical Toxicology*.

[84] Rivera C. A., Ferro C. L., Bursua A. J., Gerber B. S. (2012). Probable interaction between Lycium barbarum (goji) and warfarin. *Pharmacotherapy*.

[85] Zhang J., Tian L., Xie B. (2015). Bleeding due to a probable interaction between warfarin and Gouqizi (Lycium Barbarum L.). *Toxicology Reports*.

[86] Arroyo-Martinez Q., Sáenz M. J., Arias F. A., Acosta M. S. J. (2011). Lycium barbarum: a new hepatotoxic “natural” agent?. *Digestive and Liver Disease*.

[87] Cao Y., Ma Z. F., Zhang H., Jin Y., Zhang Y., Hayford F. (2018). Phytochemical properties and nutrigenomic implications of Yacon as a potential source of prebiotic: current evidence and future directions. *Food*.

[88] Ma Z. F., Zhang H. (2017). Phytochemical constituents, health benefits, and industrial applications of grape seeds: a mini-review. *Antioxidants*.

[89] Ravichanthiran K., Ma Z. F., Zhang H. (2018). Phytochemical profile of brown rice and its nutrigenomic implications. *Antioxidants*.

[90] Ma Z. F., Lee Y. Y. (2016). Virgin coconut oil and Its cardiovascular health benefits. *Natural Product Communications*.

[91] Zhang H., Ma Z. F. (2018). Phytochemical and pharmacological properties of *Capparis spinosa* as a medicinal plant. *Nutrients*.

